# Correlates of Cessation Success among Romanian Adults

**DOI:** 10.1155/2014/675496

**Published:** 2014-06-04

**Authors:** Dorota Kaleta, Bukola Usidame, Elżbieta Dziankowska-Zaborszczyk, Teresa Makowiec-Dąbrowska

**Affiliations:** ^1^Department of Preventive Medicine, Medical University of Łódź, 90 752 Łódź, Poland; ^2^Department of Public Policy, University of Massachusetts, Boston, MA 02125, USA; ^3^Department of Social and Preventive Medicine, Medical University of Łódź, 90 752 Łódź, Poland; ^4^Department of Work Physiology and Ergonomics, Nofer Institute of Occupational Medicine, 91 348 Łódź, Poland

## Abstract

*Background*. Tobacco smoking and its consequences are a serious public health problem in Romania. Evidence-based data on factors associated with successful smoking cessation are crucial to optimize tobacco control. The aim of the study was to determine the sociodemographic and other factors associated with smoking cessation success among adults. *Materials and Methods*. Data was from a sample of 4,517 individuals derived from the Global Adult Tobacco Survey (GATS). GATS is a cross-sectional, nationally representative household survey implemented in Romania in 2011. Data was analyzed with logistic regression. *Results*. Among females, the quit rate was 26.3% compared with 33.1% in males (*P* < 0.02). We found disparities in cessation success among the analyzed groups of respondents. Being economically active, being aged 40 and above, and having an awareness of smoking health consequences were associated with long-term quitting smoking among men, while initiating smoking at a later age increased the odds of quitting smoking among women. However, cohabitation with nonsmokers was the strongest predictor of successful cessation among both genders. *Conclusion*. Programs increasing quit rates and encourage cessation among groups less likely to quit, adopting voluntary smoke-free homes, and increasing the awareness of smoking and tobacco pollution risks are needed.

## 1. Introduction

Tobacco smoking and its adverse consequences are a serious public health problem in Romania. Smoking prevalence in 2011 was 37.4% and 16.6% among adult men and women, respectively [1]. According to the World Health Organization, in Romania, tobacco was responsible for 16% of all noncommunicable diseases (NCDs) compared with 20% of all communicable diseases [2]. The proportion of deaths attributable to tobacco was approximately 24% for men and 6% for women. Among those who died prematurely, almost one in every 4 deaths (population aged 30–44) and one in 3 deaths (population aged 45–59 years) were attributable to tobacco use.

Peto et al. estimate that smokers who die as a result of their tobacco consumption die 14 years earlier than people who never smoked [3]. Expressed in life years, it was calculated that in 2010, about 9.94 million years were lost prematurely. The estimated loss to society caused by premature deaths associated with smoking amounts to a monetized value of 517 bEUR for the EU, which corresponds to about 4.7% of the gross domestic product (GDP) [3]. Romania was hugely affected with an estimated loss of 26,611 mEUR, which corresponds to about 11.2% of the GDP. However, the losses caused by tobacco extend beyond the costs of premature deaths and also affect other aspects of society functions.

To decrease the burden of smoking-induced disease as well as related society costs, there is a need to reduce smoking. Increasing the cessation rate is considered the only high-impact strategy that can determine a significant improvement in a relatively short term [4]. There are many approaches and interventions at individual as well as population levels that have been assessed and implemented, and their effective results could serve as an example [4–9].

In regard to the above, the purpose of this study was to determine the sociodemographic and other factors (including risk awareness of smoking and environmental tobacco smoke harm or cohabitation with a smoker) associated with successful smoking cessation among Romanian adults in order to provide evidence-based data to develop well-tailored, effective tobacco control strategies.

## 2. Material and Methods

Data on smoking status, sociodemographic and other characteristics of respondents were derived from the Global Adult Tobacco Survey (GATS). Detailed methodology of the survey was described elsewhere [1, 10, 11]. Romania belongs to the EURO World Health Organization countries and it is also one of the GATS family countries that implemented survey in years 2009–2011, alongside the Russian Federation, Poland, and Ukraine. Global Adult Tobacco Survey is a cross-sectional, nationally representative household survey [11]. GATS data were collected electronically by trained pollsters during in-person interviews. The target population was noninstitutional residents aged 15 years and older. According to the GATS sample selection requirements, a two-phase sampling for GATS Romania was conducted in which a subsample of primary sampling units (PSUs) was selected from the master sample EMZOT (Multifunctional Sample on Territorial Areas) [1]. The final probability selection of the sample units was equivalent to those being selected under three-stage stratified-cluster sampling, which were selected in order to produce key indicators for the whole country, also classified by residence (urban or rural) and by gender. Of the 5,629 sampled households, 4,601 were completely filled in the household interview, and the computed household response rate was 89.9%. The household response rate was higher in rural areas than in urban areas (95.8% and 85.6%, resp.). Among individuals selected from the completely screened households, 4,517 completed the individual interview, and the computed person-level response rate was 98.4%. The total response rate was 88.5%.

Data used for current analysis is publicly available from the Global Tobacco Surveillance System (GTSS).

## 3. Study Variables

The outcome variable was successful smoking cessation for one year or longer among adults in Romania. A successful quitter was defined as regular smoker (consuming at least one cigarette per day) who had stopped smoking for at least one year prior to the interview. Those respondents who had quit smoking more recently were considered recent quitters. A current smoker was defined as someone who had smoked more than an average of one cigarette per day on a regular basis for at least one year. The ever smokers group include all the above-mentioned categories including respondents who were current, former smokers and recent quitters. Overall lifetime cessation rates or “quit rates” were calculated, as the number of former smokers divided by the number of ever smokers and multiplied by 100% [10].

The exposure variables applied for determining associations of successful cessation were the gender (male, female) and age (under 25, 25–29, 30–39, 40–49, 50–59, and 60 years and older) of the respondents. Moreover, age at smoking onset, the age at which respondents started to smoke tobacco on a regular basis, was considered (≤17, 18–20, and 21 years or over). Educational attainment was regarded as primary education, secondary education, and high education. Accordingly, occupational classification of respondents was described as economically not active (pupils, students, persons occupied with household keeping, retired, and pensioners due to disability), currently with a permanent job as employed, and currently with no permanent job as unemployed able to work and unemployed unable to work. Furthermore, respondents' place of residence was a rural or urban area. Socioeconomic circumstances, including ownership of different household items, were also evaluated. The variable called “Asset Index” was created based on summative score of possession of the following assets: functioning electricity, flush toilet, fixed telephone, cell telephone, television, radio, refrigerator, car, washing machine, computer, and internet access. The summative score was then divided into, high, medium, and low. Similar methodology has been validated elsewhere [12]. We also assessed the awareness of the negative health consequences of smoking. Respondents were categorized as aware (those who answered “yes” to the following question: do you think that tobacco smoking causes serious diseases?) and not aware (those who answered “no” and “do not know”). Similarly, awareness of the adverse health consequences of environmental tobacco smoke (ETS) exposure was determined and respondents were characterized as aware and not aware. In addition, we considered cohabitation with a smoker (yes, no).

## 4. Analysis and Statistics

The STATISTICA Windows XP version 8.0 program was used to perform the statistical analysis. Firstly, a descriptive analysis for all variables included in the study was completed. All analyses were performed separately for men and women. Logistic regression model was implemented to compare those who successfully quit (former smokers who quit ≥1 year) with those who continued to smoke (current daily smokers). We used logistic regression analyses of unweighted data to calculate the odds ratios (ORs) and the 95% confidence interval (CI) of each indicator on outcome measure. In the first stage, crude coefficients, odds ratios (OR) of the impact of odd variables on the successful smoking cessation in males and females, were calculated. This was followed by a multifactorial analysis considering the simultaneous effect of all statistically significant variables on the possibility of successful smoking cessation.

## 5. Results

The sample comprised 4,517 respondents, of which 450 subjects (336 men and 114 women) had successfully quit smoking and had not smoked for at least 1 year before the interview. The distribution of former, current, and ever smokers, recent quitters, and quit rates of the study sample by gender are available in Table 1.

Regarding age, average male ever smokers were 49.7 ± 16.3 years of age compared to 45.9 ± 14.7 years of age for female ever smokers (*P* < 0.001). Similarly, current male smokers were 44.5 ± 14.6 years of age versus 43.6 ± 13.8 years in female (*P* > 0.05). At the mean, former smokers were a bit older, 59.9 ± 14.9 years in males and 51.9 ± 15.2 years in females (*P* < 0.001). The mean age of recent quitters was 51.5 ± 12.4 and 45.5 ± 16.5 years for men and women, respectively, (*P* < 0.05). Women started smoking later than men (data not presented in the tables). Former and current male smokers started smoking by 18.8 ± 5.3 and 18.2 ± 4.5 years of age, respectively, while female former and current smokers started at 23.0 ± 7.8 (men versus women *P* < 0.001) years, respectively. We also observed a lower quit rate among women relative to men, 26.3% for women compared to 33.1% for men (*P* < 0.02). For women who successfully quit, they quit at a slightly younger age than men. The mean age of quitting for male and female former smokers was 44.5 ± 14.0 and 41.9 ± 13.5 years, respectively (*P* > 0.05). Men and women had been smoking 26.2 ± 13.8 and 19.5 ± 11.7 years, respectively, before quitting (males versus females *P* < 0.001). At the time of the interview, male and female former smokers reported mean 15.5 ± 13.5 and 9.9 ± 8.9 years, respectively, since quitting (*P* < 0.001).

During 12 months prior to the interview, 34.0% (*n* = 223) of male current smokers and 37.1% (*n* = 111) of female current smokers (*P* > 0.05) attempted to give up smoking. Almost one-third of the current male smokers, 36.0% (*n* = 227) and 33.1% (*n* = 94) of the current female smokers had no plans to quit (*P* > 0.05). Other respondents considered giving up smoking in the future.

## 6. Univariable Analysis 

The results of the univariable regression analyses are presented in Tables 2 and 3. Male and female smokers experienced the highest likelihood to quit over the age of 60 compared to those less than 25 years of age (male: OR = 29.1, 95% CI: 10.2–82.5; female: OR = 15.3, 95% CI: 3.4–69.9, resp.). Long-term quit odds substantially increased with older age groups, older than 25 years in men and age 30–39 in women (Table 2). Also men and women who started smoking late, after age 21, are more likely to quit relative to those who started smoking before they were 17 years (male: OR = 1.5, 95% CI: 1.0–2.1; female: OR = 2.6, 95% CI: 1.4–4.8, resp.).

Male and female subjects classified as economically inactive have a higher probability of successful smoking cessation relative to the unemployed people (OR = 10.9, 95% CI: 6.1–19.4; OR = 3.2, 95% CI: 1.0–10.2, resp.). Men who were aware of smoking health consequences and ETS consequences were more likely to quit smoking successfully than those who were unaware (OR = 4.5, 95% CI: 1.8–11.5; OR = 1.8, 95% CI: 1.0–3.2, resp.). Awareness of these consequences had no significant association with successful cessation among women. People living alone or with nonsmokers were significantly more likely to quit smoking successfully than those living with smokers (male smokers: OR = 16.7, 95% CI: 11.9–23.4 and female smokers: OR = 20.1, 95% CI: 11.3–35.6). Education, place of residence, and Asset Index were not significantly associated with successful smoking cessation.

## 7. Multivariable Analysis

The results of the multivariable regression analyses are presented in Tables 2 and 3. After taking all the statistically significant variables in the univariable model into account, age for smoking initiation and awareness of ETS exposure were statistically insignificant among male smokers, while age and occupational classification were statistically insignificant among female smokers.

Cohabitation with a non-smoker was the only significant predictor of long-term smoking cessation in both genders (Tables 2 and 3). The probability of quitting smoking was significantly higher among men and women living alone or with nonsmokers than those living with smokers (male: OR = 13.9, 95% CI: 9.4–20.1 and female: OR = 20.1, 95% CI: 11.1–39.1, resp.). Age, occupation, and awareness of smoking health consequences were significant predictors of smoking cessation among men. The odds of quitting increased with age among men, as male smokers over 60 are most likely to quit smoking compared to those less than 25 (OR: 15.6, 95% CI: 4.7–51.7). Similarly, men who were economically active had a higher likelihood of quitting smoking compared to unemployed male smokers (OR: 2.6, 95% CI: 1.3–5.1). Being aware of smoking health consequences also increased a man's likelihood to stop smoking (OR: 3.1, 95% CI: 1.0–9.6).

The odds of successful smoking cessation increased with age at smoking onset among women. Women who started smoking after age 21 were more likely to quit smoking compared to those who started before age 17 (OR: 2.6, 95% CI: 1.4–4.8). Age and occupation were not correlated with smoking cessation among women.

## 8. Discussion

In this study, we have evaluated the factors affecting smoking cessation success among Romanian adults.

In Romania, the lifetime quit rate was 26.3% for women and 33.1% for men which means that approximately one-third of people who have ever smoked have quit. Similar findings were derived from GATS Poland, including lower quit rates among women compared to men [10]. However, quit rates are almost two times lower when compared to more developed countries; for example, Canada had a quit rate of approximately 59% [13]. These differences show huge disproportions among middle-income and developed countries in terms of the effectiveness of the implementation of tobacco control policies including cessation measures.

Apart from gender, GATS revealed the association of successful cessation with several other sociodemographic factors among a representative sample of adult population. GATS showed that older age was strongly associated with long-term cessation in men, which is consistent with other studies [11, 14–18]. The possible interpretation of this association is that older persons engaged in smoking cessation have greater motivation, discipline, and immediate preoccupation with health or factors that can help them to succeed. It is also known that a significant percentage of older smokers already show symptoms of smoking related diseases, which may also reinforce their interest in quitting [17]. Patients at higher risk of noncommunicable diseases are screened more often and advised to quit, which may increase quit success [19]. In Romania, 82.1% of smokers had visited a healthcare provider during the previous 12 months; this represents the proportion reported being asked if they smoked by a healthcare provider [20]. Among those screened for tobacco use, 67.3% reported that their healthcare providers advised them to quit. In most GATS countries including Romania, persons aged ≥45 years were more likely to report being screened and advised to quit than those aged ≤24 years. This also partially explains the observed association between screening and quitting. Unlike men, older age was not associated with successful smoking cessation among women. This result should be considered while planning tobacco control policies and intervention approaches but also further in-depth research are needed to clarify the reasons for such differences.

Similar to other studies, we found that older age of smoking uptake was correlated with increased cessation rates among women [21–24]. Several studies reported that being older at first cigarette use likely relates to lower lifetime exposure to cigarettes, which may link to lower levels of nicotine dependence and, in turn, lead to a higher likelihood of quitting [25, 26]. Existing data shows that difficulty in quitting increased with increased nicotine dependence and the number of prior quit attempts [14, 15, 25, 27]. Breslau and Peterson have stressed that programs that postpone smoking initiation are particularly important because even if they do not prevent the uptake of smoking among all young people, they may decrease smoking prevalence in the long run by increasing the probability for successful cessation [25].

However, cohabitation with a smoker seems to be the most important factor limiting cessation success among both genders. In order to reduce ETS exposure, European Union countries including Romania implemented smoking bans in public places and worksites, while less efforts were undertaken to encourage adoption of smoke-free rules in the private settings. Smoking bans are mainly introduced to protect nonsmokers from tobacco pollution, but it also increases quitting among smokers and prevents relapse among former smokers [4]. Data from GATS suggest that the home is a very important target and an opportunity that should be utilized for increasing quit rates and help maintain cessation success among Romanian adults.

GATS also revealed disproportions in cessation success among socioeconomic groups in Romania. Another factor that we assessed was potential correlation between successful cessation tobacco use and employment in Romanian adults. We found that the odds of successfully quitting in employed men were over two times higher compared with unemployed smokers. This result confirms the previous evidence [26, 28]. The lower odds of smoking cessation among the unemployed may result from believing that smoking is a way to reduce stress which includes unemployment. Hence, there is a probable need to promote rational ways of coping with stress especially among the unemployed. Moreover, data shows that having financial difficulties, which could be the case of unemployed men, remains an important barrier to smokers achieving quit success. Further research is required to determine strong mediators of the effect of financial difficulties on successful cessation and to tailor more effective cessation programs [29–32]. But it can also happen that after cigarette price increases, economically disadvantaged smokers switch from manufactured cigarettes to hand-rolled or less expensive brands or buy illegal products and end up not giving up smoking. Nonetheless, many smokers continue the habit in spite of an increase in smoking-associated socioeconomic inequalities. This issue is because disadvantaged smokers are not motivated to quit but rather spend more money on tobacco and less on other goods, which ultimately deepens deprivation. It suggests that aside fiscal policies, other policies should be used to increase cessation among lower socioeconomic groups [10]. However, similar to results obtained from GATS Poland, this association with fiscal policies was not present in the female group [10]. This relationship may be explained by the low participation of females in the labor market, and the socioeconomic and cultural context comparable in middle income, deriving from the post-Soviet bloc European countries. The total labor force participation rate (% of total population ages 15–64) in Romania was 64.3% as of 2011. But female labor participation rate (% of female population ages 15+) in Romania was 48.6% compared to 72% in men as of 2011 [33]. On the other hand, these results should be assessed with caution because employment status may possibly change over the life span, but due to the cross-sectional nature of the study, we can only assess the occupational situation at the time of survey completion.

In contrast to other surveys, we did not observe correlation of other factors reflecting the socioeconomic position of respondents, including education and Asset Index, with long-term cessation [34]. Positive association between success in quitting and socioeconomic resources is well established; thus the lack of association between education and Asset Index should be a subject of further studies to further enlighten this issue.

Moreover, we did not find significant association between awareness of negative health consequences of environmental tobacco smoke exposure and cessation success among men or women. This might be hypothesized that some smokers declare general knowledge on these topics but do not fully acknowledge the increased risk of cancer, stroke, and heart attack due to smoking or environmental exposure to tobacco smoke. Federico et al. revealed that risk judgment among smokers appeared to be unrealistically optimistic [34]. Smokers underestimate their risk of developing lung cancer and other tobacco related diseases. Misunderstandings of smoking and ETS risks do not encourage quitting [34].

## 9. Study Strengths and Limitations 

GATS is a nationally representative survey that includes a large number of respondents, which is carefully designed and based on a standard and consistent protocol. The questionnaire of GATS Romania was adapted from the standard GATS core questionnaire while maintaining the highest standards to ensure the accuracy and quality. GATS questionnaire covers numerous potential cessation predictors. However, several limitations should still be mentioned. The questionnaire method of data collection has a number of advantages including low cost, ease of obtaining data, and quick evaluation. A lot of studies that have investigated determinants of tobacco quitting have carried out surveys within populations with high risk of cancer or cardiovascular disease. The selected demographic variables also seem to be limited to age, race, and socioeconomic status [10, 35–40]. The methodology also varies among these studies including cross-sectional studies and intervention trials [13, 14, 19, 41–44]. In GATS study, we are unable to assess the motivations for quitting, as respondents were not asked to give their reasons for quitting smoking. Based on this, we cannot compare quitting reasons from Romania with other GATS countries. We are also unable to determine the impact of previous tobacco control measures, such as tobacco tax increase or education campaigns as influences on tobacco cessation.

Using self-reported techniques to obtain data is also a potential limitation, but it has been stated not to reduce the quality of the study, as addressed in previous papers [41, 45]. A number of variables may have been omitted from the GATS questionnaire which may have improved the validity or information provided by the results. Some of these results include nicotine dependence or number of cigarettes smoked which are considered determinants of long-term quitting, use of aids or not for quitting (for those who quit for a year or longer), number of quitting attempts, marital status, and annual household net income [38, 46]. In addition, cross-sectional studies limit the results to one point in time. We are unable to draw conclusions on causality or directionality of findings. Therefore, we are unable to determine which characteristics may have changed over time. However, this study provides the most recent national evidence on the association of successful quitting with selected characteristics in Romania.

## 10. Conclusions

GATS revealed the need to focus on policies and programs to increase quit rates and to encourage cessation among groups less likely to quit, amongst the younger, unemployed, those who start smoking at a younger age, and those unaware of smoking health consequences. This may bring great health and social and economic benefits for the entire population and is essential to prevent future widening health inequalities among disadvantaged groups. Our study results emphasize the need for an increased effort to promote adopting voluntary smoke-free homes, in order to improve the quitting rates. GATS also shows that other aspects of tobacco control in Romania should be expanded to achieve higher level of compliance with the Framework Convention on Tobacco Control (FCTC) that will support quitting.

Policy makers should consider these points to set priorities and targets for tobacco cessation measures and other tobacco control actions.

## Figures and Tables

**Table 1:** Characteristics of former, current, ever smokers, recent quitters, and quit rates by gender—Global Adult Tobacco Survey Romania 2011.

Characteristic	Male *N* = 1015					Female *N* = 433				
	Former smokers *N* = 336	Recent quitters *N* = 22	Current smokers *N* = 657	Ever smokers *N* = 1015	Quit rate^A^ %	Former smokers *N* = 114	Recent quitter *N* = 18	Current smokers *N* = 301	Ever smokers *N* = 433	Quit rate^A^ %
	*N* (%)	*N* (%)	*N* (%)	*N* (%)		*N* (%)	*N* (%)	*N* (%)	*N* (%)	
Overall	336 (33.1)	22 (2.2)	657 (64.7)	1015 (100.0)	33.1	114 (26.3)	18 (4.2)	301 (69.5)	433 (100.0)	26.3
Age (years)										
<25	4 (1.2)	0 (0.0)	61 (9.3)	65 (6.4)	6.2	2 (1.8)	2 (11.1)	29 (9.6)	33 (7.6)	6.1
25–29	7 (2.1)	2 (9.1)	61 (9.3)	70 (6.9)	10.0	5 (4.4)	2 (11.1)	28 (9.3)	35 (8.1)	14.3
30–39	28 (8.3)	0 (0.0)	140 (21.3)	168 (16.5)	16.7	24 (21.1)	2 (11.1)	67 (22.3)	93 (21.5)	25.8
40–49	46 (13.7)	9 (40.9)	159 (24.2)	214 (21.1)	21.5	18 (15.8)	6 (33.3)	74 (24.6)	98 (22.6)	18.4
50–59	68 (20.2)	6 (27.3)	140 (21.3)	214 (21.1)	31.8	29 (25.4)	1 (5.6)	69 (22.9)	99 (22.9)	29.3
≥60	183 (54.5)	5 (22.7)	96 (14.6)	284 (28.0)	64.4	36 (31.6)	5 (27.8)	34 (11.3)	75 (17.3)	48.0
Missing data	0 (0.0)	0 (0.0)	0 (0.0)	0 (0.0)	—	0 (0.0)	0 (0.0)	0 (0.0)	0 (0.0)	—
Age at smoking onset										
≤17	131 (39.1)	6 (35.3)	284 (43.3)	421 (41.8)	31.1	17 (14.9)	4 (28.6)	89 (29.7)	110 (25.7)	15.5
18–20	131 (39.1)	9 (52.9)	266 (40.6)	406 (40.2)	32.3	45 (39.5)	3 (21.4)	105 (35.0)	153 (35.8)	29.4
≥21	73 (21.8)	2 (11.8)	106 (16.2)	181 (18.0)	40.3	52 (45.6)	7 (50.0)	106 (35.3)	165 (38.5)	31.5
Missing data	1 (0.3)	5 (22.7)	1 (0.2)	7 (0.7)	—	0 (0.0)	4 (22.2)	1 (0.3)	5 (1.2)	—
Education										
Primary or less	34 (10.2)	0 (0.0)	39 (5.9)	73 (7.2)	46.5	7 (6.2)	2 (11.1)	22 (7.4)	31 (7.2)	22.6
Secondary	253 (75.8)	19 (86.4)	527 (80.5)	799 (79.0)	31.7	82 (72.6)	11 (61.1)	228 (76.5)	321 (74.8)	25.5
High	47 (14.1)	3 (13.6)	89 (13.6)	139 (13.8)	33.8	24 (21.2)	5 (27.8)	48 (16.1)	77 (18.0)	31.2
Missing data	2 (0.2)	0 (0.0)	2 (0.3)	4 (0.4)	—	1 (0.9)	0 (0.0)	3 (1.0)	4 (0.9)	—
Occupational classification										
Economically not active	200 (60.1)	8 (36.4)	147 (22.4)	355 (35.1)	56.3	41 (36.0)	8 (44.4)	63 (21.1)	112 (26.1)	36.6
Employed	118 (35.4)	11 (50.0)	389 (59.3)	518 (51.2)	22.8	69 (60.5)	8 (44.4)	215 (72.2)	292 (67.9)	23.6
Unemployed, able to work	14 (4.2)	3 (13.6)	112 (17.1)	129 (12.8)	10.9	4 (3.5)	2 (11.2)	20 (6.7)	26 (6.1)	15.4
Unemployed, unable to work	1 (0.3)	0 (0.0)	8 (1.2)	9 (0.9)	11.1	0 (0.0)	0 (0.0)	0 (0.0)	0 ( 0.0)	—
Missing data	3 (0.9)	0 (0.0)	1 (0.2)	4 (0.4)	—	0 (0.0)	0 (0.0)	3 (1.0)	3 (0.7)	—
Place of residence										
Rural	144 (42.9)	9 (40.9)	282 (42.9)	435 (42.9)	33.1	33 (28.9)	4 (22.2)	104 (34.5)	141 (32.6)	23.4
Urban	192 (57.1)	13 (59.1)	375 (57.1)	580 (57.1)	33.1	81 (71.1)	14 (77.8)	197 (65.5)	292 (67.4)	27.7
Missing data	0 (0.0)	0 (0.0)	0 (0.0)	0 (0.0)	—	0 (0.0)	0 (0.0)	0 (0.0)	0 (0.0)	—
Asset Index										
High	177 (53.6)	14 (63.6)	335 (51.5)	526 (52.4)	33.6	75 (68.2)	11 (61.1)	173 (58.5)	259 (61.1)	29.0
Middle	118 (35.8)	6 (27.3)	222 (34.1)	346 (34.5)	34.1	25 (22.7)	5 (27.8)	91 (30.7)	121 (28.5)	20.7
Low	35 (10.6)	2 (9.1)	94 (14.4)	131 (13.1)	26.7	10 (9.1)	2 (11.1)	32 (10.8)	44 (10.4)	22.7
Missing data	6 (1.8)	0 (0.0)	6 (0.9)	12 (1.2)	—	4 (3.5)	0 (0.0)	5 (1.7)	9 (2.1)	—
Awareness of smoking health consequences										
Yes	328 (98.5)	21 (100.0)	612 (93.6)	961 (95.3)	34.1	111 (97.4)	18 (100.0)	285 (95.6)	414 (96.3)	26.8
No	5 (1.5)	0 (0.0)	42 (6.4)	47 (4.7)	10.6	3 (2.6)	0 (0.0)	13 (4.4)	16 (3.7)	18.7
Missing data	3 (0.9)	1 (4.5)	3 (0.5)	7 (0.7)	—	0 (0.0)	0 (0.0)	3 (1.0)	3 (0.7)	—
Awareness of smoking ETS consequences										
Yes	314 (95.2)	21 (100.0)	591 (91.5)	926 (92.9)	33.9	106 (94.6)	16 (94.1)	272 (91.6)	394 (92.5)	26.9
No	16 (4.9)	0 (0.0)	55 (8.5)	71 (7.1)	22.5	6 (5.4)	1 (5.9)	25 (8.4)	32 (7.5)	18.7
Missing data	6 (1.8)	1 (4.5)	11 (1.7)	18 (1.8)	—	2 (1.8)	1 (4.6)	4 (1.3)	7 (1.6)	—
Cohabitation with a smoker										
Yes	58 (17.4)	3 (13.6)	511 (77.8)	572 (56.5)	10.1	19 (16.7)	3 (16.7)	241 (80.1)	263 (60.7)	7.2
No	276 (82.6)	19 (86.4)	146 (22.2)	441 (43.5)	62.6	95 (83.3)	15 (83.3)	60 (19.9)	170 (39.3)	55.9
Missing data	2 (0.6)	0 (0.0)	0 (0.0)	2 (0.2)	—	0 (0.0)	0 (0.0)	0 (0.0)	0 (0.0)	

^A^Quit rates calculated as a number of former smokers and divided by denominator = number of ever smokers × 100.

ETS: environmental tobacco smoke.

**Table 2:** Odds ratios (OR) and 95% confidence intervals (CI) for successful smoking cessation to selected sociodemographic and other characteristics in men—Global Adult Tobacco Survey Romania 2011.

Variable	Total *N* = 993	Former smoker *N* = 336			Current smoker *N* = 657			Univariable logistic regression		Multivariable logistic regression^a^	
	*N* %	*N*	%	95% CI	*N*	%	95% CI	OR	95% CI	OR	95% CI
Age (years)											
<25	65 (6.4)	4	6.2	0.4–12.1	61	93.8	87.9–99.6	1.00	Reference	1.00	Reference
25–29	68 (6.8)	7	10.3	3.1–17.5	61	89.7	82.5–96.9	1.75	0.49–6.29	1.79	0.45–7.18
30–39	168 (16.9)	28	16.7	11.1–22.3	140	83.3	77.6–88.9	3.05*	1.02–9.08	3.02	0.92–9.95
40–49	205 (20.6)	46	22.4	16.7–28.1	159	77.6	71.9–83.3	4.41**	1.52–12.80	4.34*	1.35–13.89
50–59	208 (20.9)	68	32.7	26.3–39.1	140	67.3	60.9–73.7	7.41***	2.58–21.24	5.30**	1.68–16.78
≥60	279 (28.1)	183	65.6	60.0–71.2	96	34.4	28.8–40.0	29.07***	10.25–82.48	15.60***	4.71–51.67
Age at smoking onset											
≤17	415 (41.9)	131	31.6	27.1–36.1	284	68.4	63.9–72.9	1.00	Reference	1.00	Reference
18–20	397 (40.1)	131	33.0	28.4–37.6	266	67.0	62.4–71.6	1.07	0.79–1.43	1.01	0.67–1.52
≥21	179 (18.1)	73	40.8	33.6–48.0	106	59.2	52.0–66.4	1.49*	1.04–2.15	1.05	0.63–1.76
Education											
Primary or less	73 (7.4)	34	46.6	35.2–58.0	39	53.4	42.0–64.8	1.65	0.92–2.95		
Secondary	780 (78.8)	253	32.4	29.1–35.7	527	67.6	64.3–70.9	0.91	0.62–1.34		
High	136 (13.8)	47	34.6	26.6–42.6	89	65.4	47.4–73.4	1.00	Reference	1.00	Reference
Occupational classification											
Economically not active	347 (35.1)	200	57.6	52.4–62.8	147	42.4	37.2–47.6	10.88***	6.10–19.41	3.66***	1.71–7.86
Employed	507 (51.3)	118	23.3	19.6–27.0	389	76.7	73.0–80.4	2.43**	1.36–4.32	2.59**	1.32–5.09
Unemployed	135 (13.6)	15	11.1	5.8–16.4	120	88.9	83.6–94.2	1.00	Reference	1.00	Reference
Place of residence											
Rural	426 (42.9)	144	33.8	29.3–38.3	282	66.2	61.7–70.7	1.00	Reference	1.00	Reference
Urban	567 (57.1)	192	33.9	30.0–37.8	375	66.1	62.2–70.0	1.02	0.77–1.31		
Asset Index											
High	512 (52.2)	177	34.6	30.5–38.7	335	65.4	61.3–69.5	1.44	0.76–2.73		
Middle	340 (34.7)	118	34.7	29.6–39.8	222	65.3	60.2–70.4	1.39	0.71–2.70		
Low	129 (13.2)	35	27.1	19.4–34.8	94	72.9	65.2–80.6	1.00	Reference	1.00	Reference
Awareness of smoking health consequences											
Yes	940 (95.2)	328	34.9	31.9–37.9	612	65.1	62.1–68.1	4.50**	1.76–11.51	3.14*	1.03–9.62
No	47 (4.8)	5	10.6	1.8–19.4	42	89.4	80.6–98.2	1.00	Reference	1.00	Reference
Awareness of smoking ETS consequences											
Yes	905 (92.7)	314	34.7	31.6–37.8	591	65.3	62.2–68.4	1.83*	1.03–3.24	1.81	0.81–4.01
No	71 (7.3)	16	22.5	12.8–32.2	55	77.5	67.8–87.2	1.00	Reference	1.00	Reference
Cohabitation with a smoker											
Yes	569 (57.4)	58	10.2	7.7–12.7	511	89.8	87.3–92.3	1.00	Reference	1.00	Reference
No	422 (42.6)	276	65.4	60.9–69.9	146	34.6	30.1–39.1	16.66***	11.87–23.37	13.78***	

ETS: environmental tobacco smoke.

^a^Fully adjusted model including all statistically significant variables.

*P ≤ 0.05.

**P ≤ 0.01.

***P ≤ 0.001.

**Table 3:** Odds ratios (OR) and 95% confidence intervals (CI) for successful smoking cessation to selected sociodemographic and other characteristics in women—Global Adult Tobacco Survey Romania 2011.

Variable	Total *N* = 415	Former smoker *N* = 114			Current smoker *N* = 301			Univariable logistic regression		Multivariable logistic regression^a^	
	*N* %	*N*	%	95% CI	*N*	%	95% CI	OR	95% CI	OR	95% CI
Age (years)											
<25	31 (7.5)	2	6.5	0.1–15.2	29	93.5	84.8–99.9	1.00	Reference	1.00	Reference
25–29	33 (9.9)	5	15.2	3.0–27.4	28	84.8	82.6–97.0	2.59	0.46–14.53	2.71	0.37–19.87
30–39	91 (21.9)	24	26.4	17.3–35.5	67	73.6	64.5–82.7	5.19*	1.16–23.56	5.57	0.96–32.22
40–49	92 (22.2)	18	19.6	11.5–27.7	74	80.4	72.3–88.5	3.53	0.77–16.24	2.53	0.43–14.92
50–59	98 (23.6)	29	29.6	20.6–38.6	69	70.4	61.4–79.4	6.09*	1.36–27.38	3.55	0.63–20.09
≥60	70 (16.9)	36	51.4	39.7–63.1	34	48.6	36.9–60.3	15.35***	3.37–69.92	4.73	0.71–31.35
Age at smoking onset											
≤17	106 (25.6)	17	16.0	9.0–23.0	89	84.0	77.0–91.0	1.00	Reference	1.00	Reference
18–20	150 (36.2)	45	30.0	22.7–37.3	105	70.0	62.7–77.3	2.24*	1.20–4.20	2.70*	1.21–6.02
≥21	158 (38.2)	52	32.9	25.6–40.2	106	67.1	59.8–74.4	2.57**	1.38–4.76	2.13	0.93–4.90
Education											
Primary or less	29 (7.1)	7	24.1	8.5–39.7	22	75.9	60.3–91.5	0.64	0.24–1.70		
Secondary	310 (75.4)	82	26.5	21.6–31.4	228	73.5	68.6–78.4	0.72	0.41–1.25		
High	72 (17.5)	24	33.3	22.4–44.2	48	66.7	55.8–77.6	1.00	Reference	1.00	Reference
Occupational classification											
Economically not active	104 (25.2)	41	39.4	30.0–48.8	63	60.6	51.2–70.0	3.25*	1.03–10.25	0.79	0.21–3.01
Employed	284 (68.9)	69	24.3	19.3–29.3	215	75.7	70.7–80.7	1.60	0.53–4.87	0.77	0.15–3.96
Unemployed	24 (5.9)	4	16.7	1.8–31.6	20	83.3	68.4–98.2	1.00	reference	1.00	Reference
Place of residence											
Rural	137 (33.0)	33	24.1	16.9–31.3	104	75.9	68.7–83.1	1.00	Reference	1.00	Reference
Urban	278 (67.0)	81	29.1	23.8–34.4	197	70.9	65.6–76.2	1.30	0.81–2.07		
Asset Index											
High	248 (61.1)	75	30.2	24.5–35.9	173	69.8	64.1–75.5	1.50	0.46–4.88		
Middle	116 (28.6)	25	21.6	14.1–29.1	91	78.4	70.9–85.9	0.83	0.23–3.00		
Low	42 (10.3)	10	23.8	10.9–36.7	32	76.2	63.3–79.1	1.00	Reference	1.00	Reference
Awareness of smoking health consequences											
Yes	396 (96.1)	111	28.0	23.6–32.4	285	72.0	67.6–76.4	1.62	0.65–4.08		
No	16 (3.8)	3	18.8	0.1–37.9	13	81.2	62.1–99.9	1.00	Reference	1.00	Reference
Awareness of smoking ETS consequences											
Yes	378 (92.4)	106	28.0	23.5–32.5	272	72.0	67.5–76.5	1.69	0.47–6.06		
No	31 (7.6)	6	19.4	5.5–33.3	25	80.6	66.7–94.5	1.00	Reference	1.00	Reference
Cohabitation with a smoker											
Yes	260 (62.6)	19	7.3	4.1–10.5	241	92.7	89.5–95.9	1.00	Reference	1.00	Reference
No	155 (37.4)	95	36.8	29.2–44.4	60	63.2	55.6–70.8	20.08***	11.34–35.56	20.88***	11.15–39.11

ETS: environmental tobacco smoke.

^a^Fully adjusted model including all statistically significant variables.

*P ≤ 0.05.

**P ≤ 0.01.

***P ≤ 0.001.
